# Transplantation of Rat Mesenchymal Stem Cells Overexpressing Hypoxia-Inducible Factor 2*α* Improves Blood Perfusion and Arteriogenesis in a Rat Hindlimb Ischemia Model

**DOI:** 10.1155/2017/3794817

**Published:** 2017-11-07

**Authors:** Weifeng Lu, Xiaoli Chen, Yi Si, Shichai Hong, Zhengyu Shi, Weiguo Fu

**Affiliations:** ^1^Department of Vascular Surgery, Zhongshan Hospital of Xiamen University, Xiamen, China; ^2^Cancer Research Center, Medical College of Xiamen University, Xiamen, China; ^3^Department of Cardiovascular Surgery, Xin Hua Hospital affiliated to Shanghai Jiao Tong University School of Medicine, Shanghai, China; ^4^Department of Vascular Surgery, Zhongshan Hospital of Fudan University, Shanghai, China

## Abstract

Mesenchymal stem cells (MSCs) have been increasingly tested in cell-based therapy to treat numerous diseases. Genetic modification to improve MSC behavior may enhance posttransplantation outcome. This study aims to test the potential therapeutic benefits of rat bone marrow MSCs overexpressing hypoxia-inducible factor 2*α* (rMSCs^HIF-2*α*^) in a rat hindlimb ischemia model. PBS, rMSCs, or rMSCs^HIF-2*α*^ were injected into rat ischemic hindlimb. Compared with the injection of PBS or rMSCs, transplantation of rMSCs^HIF-2*α*^ significantly improved blood perfusion, increased the number of vessel branches in the muscle of the ischemic hindlimb, and improved the foot mobility of the ischemic hindlimb (all *P* < 0.05). rMSC^HIF-2*α*^ transplantation also markedly increased the expression of proangiogenic factors VEGF, bFGF, and SDF1 and Notch signaling proteins including DII4, NICD, Hey1, and Hes1, whereas it reduced the expression of proapoptotic factor Bax in the muscle of the ischemic hindlimb. Overexpression of HIF-2*α* did not affect rMSC stemness and proliferation under normoxia but significantly increased rMSC migration and tube formation in matrigel under hypoxia (all *P* < 0.05). RMSCs^HIF-2*α*^ stimulated endothelial cell invasion under hypoxia significantly (*P* < 0.05). Genetic modification of rMSCs via overexpression of HIF-2*α* improves posttransplantation outcomes in a rat hindlimb ischemia model possibly by stimulating proangiogenic growth factors and cytokines.

## 1. Background

Mesenchymal stem cells (MSCs) are multipotent stem cells and capable to differentiate into a variety of cell types, such as osteocytes, adipocytes, and chondrocytes. Because MSCs can be isolated from various tissues and have an immunoregulatory feature to reduce the risk of host rejection, they are becoming a promising cell source for cell-based therapy [1]. The potential therapeutic benefits of MSCs have been intensively investigated in numerous animal disease models, including neurodegenerative disease, autoimmune disease, and cardiovascular disease [1]. Despite the efforts, the therapeutic potential of MSCs may still be hindered by their behavior after transplantation [2]. For instance, the proliferation rate of MSCs is relatively low [3, 4]; oxidative stress during isolation may induce apoptosis [5, 6].

Tissues containing MSCs usually have low oxygen pressure. For instance, the oxygen pressure is 1–7% and 1.5–5% in bone marrow and female reproductive tissues, respectively [7, 8]. Ma and colleagues have demonstrated that oxygen pressure in MSC niche is approximately 2–8% [9]. Thus, normoxic in vitro culturing condition can induce oxidative stress in MSCs, leading to apoptosis [10, 11]. Accumulating in vivo and in vitro evidence supports that hypoxic preconditioning can prevent apoptosis and enhance MSC survival [12–15]. In addition to preconditioning, genetic modification to improve MSC survival has also been investigated recently [2]. Hypoxia-inducible factors (HIFs), which are hypoxia-induced transcription factors, regulate the expression of molecules that play critical roles in inducing angiogenesis, preventing apoptosis, and stimulating migration of cells under hypoxia condition [16]. Overexpression of HIF-1*α* in human bone marrow MSCs increases the production of vascular endothelial growth factor (VEGF) and stem cell factor, stimulates c-Met expression, and enhances MSC tube formation [17, 18]. However, MSCs overexpressing HIF-1*α* have not been tested in animal models, and the effect of MSCs overexpressing HIF-1*α* on posttransplantation outcome remains unexplored.

HIF-2*α* is also an essential transcription factor induced by hypoxia. HIF-1*α* and HIF-2*α* have distinct roles in hypoxia response and regulate different target genes [19, 20]. In renal carcinoma cells, HIF-1*α* mainly mediates hypoxia-induced VEGF upregulation, whereas HIF-2*α* regulates a broader range of target genes, including VEGF, glucose transporter-1, urokinase plasminogen activator surface receptor, and plasminogen activator inhibitor-1 [21]. Overexpression of HIF-2*α* in MSCs may also exert beneficial effects on MSCs to improve posttransplantation outcome. Here, this study aims to test this hypothesis. In the current study, rat bone marrow MSCs (rMSCs) were stably transduced with lentivirus harboring HIF-2*α*. The therapeutic potential of rMSCs overexpressing HIF-2*α* (rMSCs^HIF-2*α*^) in a rat model of hindlimb ischemia was also examined.

## 2. Materials and Methods

### 2.1. Animals

Male Sprague-Dawley rats (6–8 weeks old) were purchased from the Experimental Animal Center of Xiamen University (Xiamen, China). The rats were housed in the Animal Care Center of Xiamen University according to the standard protocol for experimental animals. The protocol for handling and experimenting of rats has been approved by the Institutional Review Board of Xiamen University.

### 2.2. Cell Culture

Rat mesenchymal stem cells (rMSCs) were purchased from Cyagen Biosciences Inc., China, and cultured in F344 Rat Mesenchymal Stem Cell Basal media supplemented with 10% Mesenchymal Stem Cell-Qualified Fetal Bovine Serum (Cyagen Biosciences Inc., China), 1% penicillin-streptomycin, and 1% glutamine (Thermo Fisher Scientific, Waltham, MA, USA). Human embryonic kidney 293T (HEK-293T) cells were purchased from American Type Culture Collection (ATCC) (Manassas, VA, USA) and cultured in Dulbecco's modified Eagle's media (DMEM) supplemented with 10% fetal bovine serum and 1% penicillin-streptomycin solution (Thermo Fisher Scientific, Waltham, MA, USA) at 37°C and 5% CO_2_. Endothelial cells was purchased from Sciencell Research Laboratories (San Diego, CA, USA).

### 2.3. Construction of Recombinant Lentivirus Harboring HIF-2*α*

Rat HIF-2*α* (NM_023090) DNA with the flanking sequences of the restrictive endonuclease BamHI and AgeI sites was synthesized by New England Biolabs Inc., USA. The lentivirus vector pGC-LV-GV287 (Shanghai Genechem Ltd., Shanghai, China) was digested with BamHI and AgeI and ligated with the HIF-2*α* DNA sequence. Recombinant pGC-LV-GV287-HIF-2*α* was confirmed by DNA sequencing. Lentivirus packaging system (Shanghai Genechem Ltd., Shanghai, China) and HEK-293T cells were used to prepare the lentivirus overexpressing HIF-2*α*. The recombinant virus preparation was tittered, and the expression of HIF-2*α* in HEK-293T cells was confirmed by both RT-PCR and Western blot.

### 2.4. Transduction of rMSCs with the Recombinant Lentivirus Harboring HIF-2*α*

RMSCs at exponential growth stage were seeded in 96-well tissue culture plates at the density of 1 × 10^4^ cells per well. After the rMSCs grew to 60% confluency, the culture media were removed and the cells were then incubated with fresh culture media containing recombinant lentivirus harboring HIF-2*α* for 12 hours. The cells were then washed with PBS and incubated with fresh complete media for another 48 hours. Transduction efficiency was estimated by the percentage of rMSCs showing green fluorescence. RMSCs were transduced with the recombinant virus at multiplicity of infection (MOI) 0, 25, 50, or 100. The MOI that produced >80% transduction efficiency was used for the rest of the study.

### 2.5. Western Blot

RMSCs overexpressing HIF-2*α* (rMSCs^HIF-2*α*^) were cultured to 90% confluency and total protein was extracted. The total protein was separated by SDS-PAGE and transferred to a PVDF membrane. The membrane was blocked in Tris-buffered saline containing 1% Tween 20 (TBST) and 5% skim milk at room temperature for 2 hours and then incubated with rabbit anti-rat HIF-2*α* antibody (1 : 500, ab179825, Abcam, USA) at 4°C overnight. After 3 washes with TBST, the membrane was incubated with the secondary antibody goat anti-rabbit IgG (1: 10000, Sigma-Aldrich, USA) at room temperature for 2 hours. After 5 washes, signals of the target protein were developed using the enhanced chemiluminescence substrates (Pierce ECL, Thermo Fisher Scientific). Western blot was also performed to analyze the expression of cytokines in the muscles of ischemic limb. Rats were euthanized 14 days after the surgery for establishment of hindlimb ischemia and cell transplantation and muscle tissue of the ischemic limb was collected. The tissue was homogenized, and total protein was extracted, separated on SDS-PAGE, and transferred to a PVDF membrane. The membrane was probed with rabbit anti-rat HIF-2*α*, delta-like ligand 4 (DII4), Notch intracellular domain (NICD, 1 : 500, ab52301, Abcam), Hey1 (1 : 500, ab154077, Abcam), Hes1 (1 : 500, ab108937, Abcam), VEGFR2 (1 : 500, ab45010, Abcam), VEGF-A (1 : 500, ab115961, Abcam), NRP-1 (1 : 500, ab190598, Abcam), and Tie-2 (1 : 500, ab137786, Abcam). *β*-Actin (1: 5000, A5441, Sigma-Aldrich, USA) was used as the internal control.

### 2.6. Verification of the Stemness of rMSCs Overexpressing HIF-2*α*

To verify the stemness of rMSCs^HIF-2*α*^, we determined the expression of cell surface CD29 and CD45 by flow cytometry and tested the multiple differentiation potential of rMSCs^HIF-2*α*^. For flow cytometry, rMSCs^HIF-2*α*^ were harvested, incubated with R-phycoerythrin (PE) hamster anti-rat CD29 (1 : 100, 562154, BD Biosciences, San Jose, CA, USA) and PE mouse anti-rat CD45 (1 : 50, sc-70696, Santa Cruz Biotechnology Inc., Dallas, TX, USA) for one hour, and analyzed on a flow cytometer (BD LSR II Flow Cytometer System; BD Biosciences, San Jose, USA). RMSCs^HIF-2*α*^ were induced to differentiate into adipocytes, osteoblasts, or chondrocytes by incubating with adipocyte, osteoblast, or chondrocyte differentiation induction medium (Cyagen Biosciences Inc., USA). Lipid expression and production in the differentiated fat cells were determined by oil red O staining (Oil Red O kit was from BioVision Inc., Milpitas, CA, USA) according to the protocol from the kit. Calcium deposit in the differentiated osteoblasts was stained with alizarin red S according to the protocol in the alizarin red S staining kit (Sciencell Research laboratories, San Diego, CA, USA). Glycosaminoglycan matrix in the differentiated chondrocytes was stained with toluidine blue (toluidine blue stain kit was from GeneCopoeia Inc., Rockville, MD, USA) according to the protocol provided by the manufacturer.

### 2.7. Rat Hindlimb Ischemia Model and rMSC Transplantation

A total of 32 rats were randomized into the following 4 groups (8 per group): sham, PBS, rMSC, and rMSC^HIF-2*α*^ groups. The rats were anesthetized with pentobarbital sodium (50 mg/kg). The hindlimb ischemia model was established by the resection of the left common iliac artery. The distal portion of the left common iliac artery, the femoral artery, and the associated side braches were also dissected and excised. In the sham group, rats underwent anesthesia, and the left common iliac artery, the femoral artery, and the associated branches were dissected but without ligation or excision. After suture, postoperative analgesia with buprenorphine (0.05–0.1 mg/kg intramuscularly) was given as needed, and the rats were maintained in separate cages (one per cage). Immediately after the resection, 5 × 10^6^ rMSCs, rMSCs^HIF-2*α*^, or PBS in 200 *μ*L were injected into the ischemic thigh muscle with a 26-gauge needle at 6 different positions in the tibialis anterior muscle and gastrocnemius. The mobility damage and neoangiogenesis of the ischemic thigh were assessed 7, 14, 21, and 28 days after the transplantation.

### 2.8. Assessment of the Perfusion and Mobility Damage of the Ischemic Thigh

Blood perfusion was assessed with a laser Doppler perfusion image (LDPI) analyzer (Moor Instruments, Axminster, UK) 7, 14, 21, and 28 days after the transplantation. A dark-blue color on the images represents low or no blood perfusion; a red color represents high blood flow. Both the ischemic and the nonischemic limbs were scanned twice, and the average blood flow values were calculated. The LDPI index was then calculated as the ratio of the perfusion of ischemic hindlimb to the perfusion of the nonischemic hindlimb [22]. The mobility damage of the ischemic limb was evaluated before the surgery and 4 weeks after the surgery according to the following scoring criteria: score 3 represents that the ischemic limb was dragged during crawling movement and showed ischemic gangrene; score 2 represents that the ischemic limb showed mild dragging and moderate gangrene but without reflex response; score 1 represents that the ischemic limb showed good reflex response and mild gangrene; and score 0 represents no obvious difference in the ischemic and the nonischemic limbs.

### 2.9. Evaluation of Capillary Density and Neoangiogenesis

Rats were euthanized 28 days after the transplantation. A total of 4 pieces of tissue from the adductor and semimembranous muscles were removed and used to prepare frozen tissue sections. The frozen tissue sections were stained for alkaline phosphatase using a fast BCIP/NBT solution (Sigma, St. Louis, MI, USA). Five observation fields were randomly selected from each tissue section. Capillary density was calculated as the ratio of the number of capillaries to the number of muscle fibers [23]. Neoangiogenesis in the ischemic limb was evaluated by microangiography according to the previous description [24]. Microangiography was performed at the Beamline BL13W1, the X-ray imaging, and biomedical application station of the Shanghai Synchrotron Radiation Facility (SSRF) in China.

### 2.10. ELISA

Blood was collected from the rats on days 0, 7, 14, 21, and 28 after the surgery. Serum angiopoietin 1 (Ang1) and Ang2 levels were determined using the ELISA kit (ab209883, Abcam).

### 2.11. Cell Proliferation Assay

The proliferation of rMSCs^HIF-2*α*^ and rMSCs was determined by Alamar Blue assay. Cells at the density of 1500 cells/100 *μ*L per well were seeded in 96-well plates. The Alamar Blue solution (10 *μ*L, Thermo Fisher Scientific) was then added after 1, 2, 3, 4, and 5 days of culturing. The cells were incubated with Alamar Blue solution at 37°C for 4 hours and then 50 *μ*L of 3% SDS was added to stop the reaction. The absorbance at 540–570 nm was determined in a plate reader (Bio-Rad, USA).

### 2.12. Migration Assay

Scratch assay was conducted to determine the migration of rMSCs^HIF-2*α*^ and rMSCs under normoxia (21% O_2_) and hypoxia (5% O_2_) conditions. Cells were seeded in 6-well plates at the density of 1 × 10^6^ cells/well. After cells grew to confluency, a pipet tip was used to scratch the center of a well. The scratched wells were washed with serum-free media 3 times and incubated in low-serum media (5% FBS) for 48 hours. Phase contrast images of the scratched wells were obtained after 0, 8, 16, 24, and 48 hours of incubation. The distance of the gap was measured, and percentage of wound closure was calculated.

### 2.13. Invasion of Endothelial Cells

Transwell chamber system (BD Bioscience, USA) was used to establish a coculture of endothelial cells and rMSCs. The membrane of transwell chambers has a pore size of 8 *μ*m and coated with Matrigel (10 mg/mL, BD Bioscience, USA). Endothelial cells were seeded in the upper transwell chamber at 1 × 10^6^ cells/well, and rMSCs or rMSCs^HIF-2*α*^ were cultured in the lower chamber at 1 × 10^6^ cells/well. The coculture was cultured at 37°C under normoxia or hypoxia condition for 12 hours. Endothelial cells inside the chambers were removed with cotton swaps, and the endothelial cells that passed through the pores were fixed and stained with crystal violet. Six observation fields were randomly selected from each membrane and the number of endothelial cells was counted. The average number of cells was used.

### 2.14. In Vitro Tube Formation

RMSCs or rMSCs^HIF-2*α*^ were seeded in 12-well plates coated with Matrigel (10 mg/mL, Becton Dickinson) at 1 × 10^6^ cells/well. After 24 hours of incubation under normoxia or hypoxia condition, tube formation was examined under a phase-contrast microscope (Olympus, Japan).

### 2.15. Statistical Analyses

Data are presented as mean ± standard deviation (SD). Student's *t*-test was used for 2-group comparison and one-way ANOVA was performed for multiple group comparison. *P* value was 2-sided and *P* < 0.05 was considered statistically significant. The statistical analysis software SPSS 16.0 (SPSS Institute, Chicago, IL) was used.

## 3. Results

### 3.1. Transplantation of rMSCs^TIF-2*α*^ Improved Blood Perfusion and Increased the Number of Vessel Branches of Ischemic Hindlimb at Higher Extent Than rMSC Transplantation

**Figure 1:**
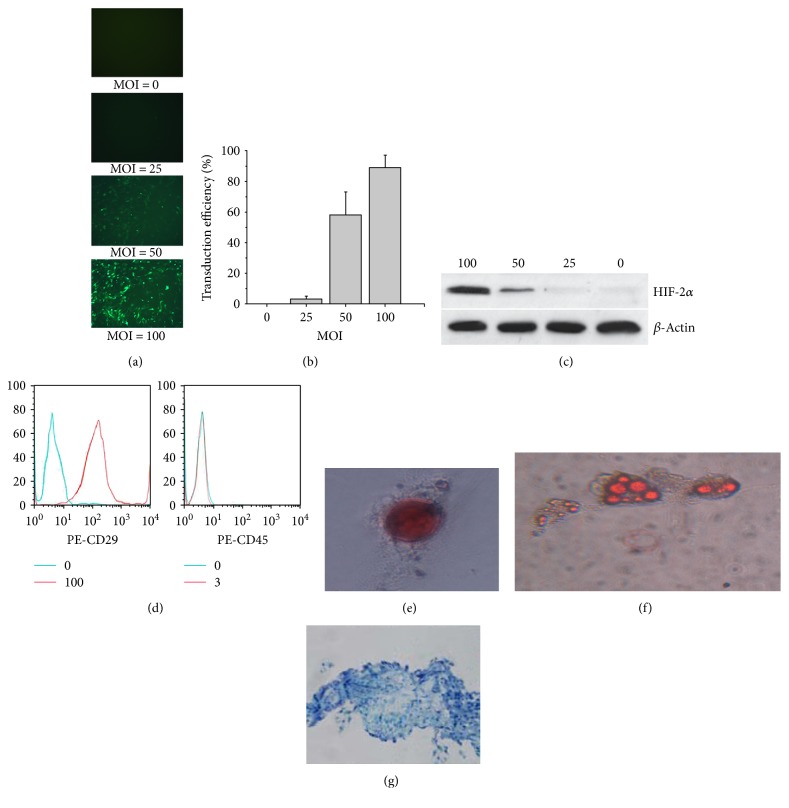
HIF-2*α* was overexpressed in rMSCs and rMSCs^HIF-2*α*^ showed stem cell characteristics. (a) Representative fluorescence images showing rMSCs transduced with 0, 25, 50, or 100 MOI recombinant lentivirus expressing HIF-2*α*. The images were collected 48 hours after transduction. (b) The dose of MOI 100 resulted in >80% transduction efficiency. (c) Representative image of Western blot showing HIF-2*α* expression in rMSCs transduced with different doses of recombinant lentivirus. (d) Representative image of flow cytometry analysis showing abundant expression of CD29 and absence of CD45 on rMSCs^HIF-2*α*^. (e) Alizarin red S staining shows calcium deposit in osteoblasts differentiated from rMSCs^HIF-2*α*^. (f) Adipocytes differentiated from rMSCs^HIF-2*α*^ shows positive oil red O staining for lipid droplets. (g) Toluidine blue staining shows positive glycosaminoglycan matrix production from chondrocytes differentiated from rMSCs^HIF-2*α*^.

Expression of HIF-2*α* from recombinant lentivirus overexpressing HIF-2*α* was confirmed by both RT-PCR and Western blot (Supplementary Figure 1 available online at https://doi.org/10.1155/2017/3794817). RMSCs were transduced with 25, 50, or 100 MOI of the recombinant lentivirus overexpressing HIF-2*α* (Figure 1(a)), and 100 MOI resulted in >80% transduction efficiency (Figure 1(b)) and abundant HIF-2*α* protein expression in rMSCs (Figure 1(c)). The rMSCs overexpressing HIF-2*α* (rMSCs^HIF-2*α*^) was charactered by positive expression of CD29 and negative expression of CD45 (Figure 1(d)) and was further verified by testing the multiple differentiation potential of rMSCs^HIF-2*α*^. RMSCs^HIF-2*α*^ were successfully induced to differentiate into osteoblasts showing calcium deposit (Figure 1(e)), adipocytes expressing lipid droplets (Figure 1(f)), and chondrocytes producing glycosaminoglycan matrix (Figure 1(g)).

**Figure 2:**
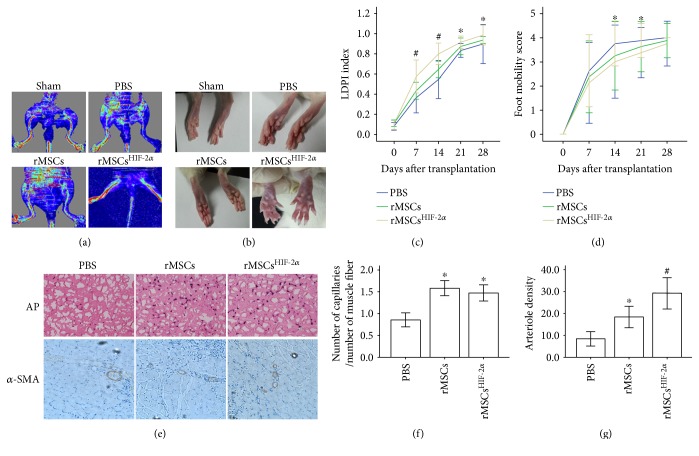
Transplantation of rMSCs^HIF-2*α*^ improved blood perfusion and increased neoangiogenesis. (a) Representative LDP images of the ischemic and nonischemic limbs. The images were collected 14 days after the transplantation. (b) Representative photos of the ischemic and nonischemic limbs. The photos were obtained 14 days after the transplantation. The sham group photo shows normal rat feet; the PBS group photo shows a loss of the big toe in the ischemic foot; the rMSC group photo shows gangrene in the ischemic foot; the rMSCs^HIF-2*α*^ group photo shows relatively normal rat feet. (c) The rMSCs^HIF-2*α*^ group showed significantly higher LDPI index than the rMSC and PBS groups. LDPI index was calculated as the ratio of the perfusion in the ischemic limb to the perfusion in the nonischemic limb. ∗ indicates significant difference of the rMSCs^HIF-2*α*^ group versus rMSC and PBS groups, *P* < 0.05. # indicates significant difference of the rMSCs^HIF-2*α*^ group versus rMSC and PBS groups, *P* < 0.01. (d) The rMSCs^HIF-2*α*^ group showed significantly lower mobility damage score of the ischemic limb than the PBS groups. ∗ indicates significant difference of the rMSCs^HIF-2*α*^ group versus the PBS group, *P* < 0.05. (e) Representative images of staining for alkaline phosphatase and *α*-smooth muscle actin (SMA). (f) Capillary density in the rMSCs and rMSCs^HIF-2*α*^ groups was significantly higher than that in the PBS group. ∗ indicates significant difference of the indicated groups versus PBS group, *P* < 0.05. (g) Arteriole density in the rMSCs and rMSCs^HIF-2*α*^ groups was significantly higher than that in the PBS group. ∗ indicates significant difference of the indicated groups versus PBS group, *P* < 0.05. # indicates significant difference of the indicated group versus PBS group, *P* < 0.01.

Transplantation of rMSCs^HIF-2*α*^ into the rat ischemic limb improved blood perfusion and mobility of the ischemic limb. LDPI showed that blood perfusion was significantly improved 14 days after the transplantation of rMSCs^HIF-2*α*^ (Figure 2(a)). Compared with the rats in the rMSCs and PBS groups, the rats injected with rMSCs^HIF-2*α*^ showed significantly higher blood perfusion in the ischemic limb after the transplantation (*P* < 0.05, Figure 2(c)). In addition, gangrene developed in the ischemic limb of the rats injected with PBS, whereas the ischemic limb of the rats transplanted with rMSCs or rMSCs^HIF-2*α*^ presented less gangrene or no gangrene 14 days after the transplantation (Figure 2(b)). The mobility of the ischemic limb in the rats with rMSCs^HIF-2*α*^ transplantation was significantly improved compared with the rats injected with PBS on days 14 and 21 after the transplantation (*P* < 0.05, Figure 2(d)).

**Figure 3:**
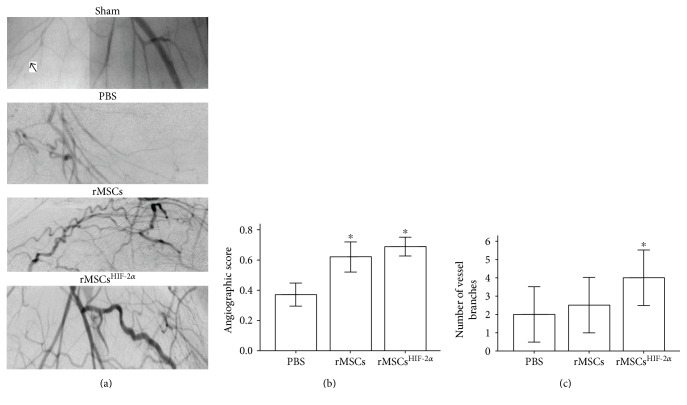
Transplantation of rMSCs^HIF-2*α*^ increased the number of vessel branches. (a) Representative images of synchrotron radiation microangiography. (b) Angiographic score in the rMSC and rMSC^HIF-2*α*^ groups was significantly higher than that in the PBS group. ∗ indicates significant difference of the indicated groups versus PBS group, *P* < 0.05. (c) The number of vessel branches in the rMSC^HIF-2*α*^ group was significantly higher than that in the rMSCs and PBS groups. ∗ indicates significant difference of the indicated group versus rMSCs and PBS groups, *P* < 0.05.

The beneficial effect of rMSC^HIF-2*α*^ transplantation on the ischemic limb appeared to be associated with an increase in neoangiogenesis. The muscle tissue in the rMSC and rMSC^HIF-2*α*^ groups showed significantly higher capillary density (the ratio of the number capillaries to the number of muscle fibers, *P* < 0.05, Figures 2(e) and 2(f)) and higher arteriole density (Figure 2(g)) than the muscle tissue in the PBS group. Synchrotron radiation microangiography further confirmed that arteriogenesis in the rMSC and rMSC^HIF-2*α*^ groups increased significantly compared with that in the PBS group (*P* < 0.05, Figures 3(a) and 3(b)). Notably, the number of vessel branches was significantly higher in the rMSC^HIF-2*α*^ group than in the rMSCs and PBS groups on day 28 after the transplantation (*P* < 0.05, Figure 3(c)).

### 3.2. The Molecular Mechanism Underlying the Beneficial Effect of rMSC^HIF-2*α*^ Transplantation

**Figure 4:**
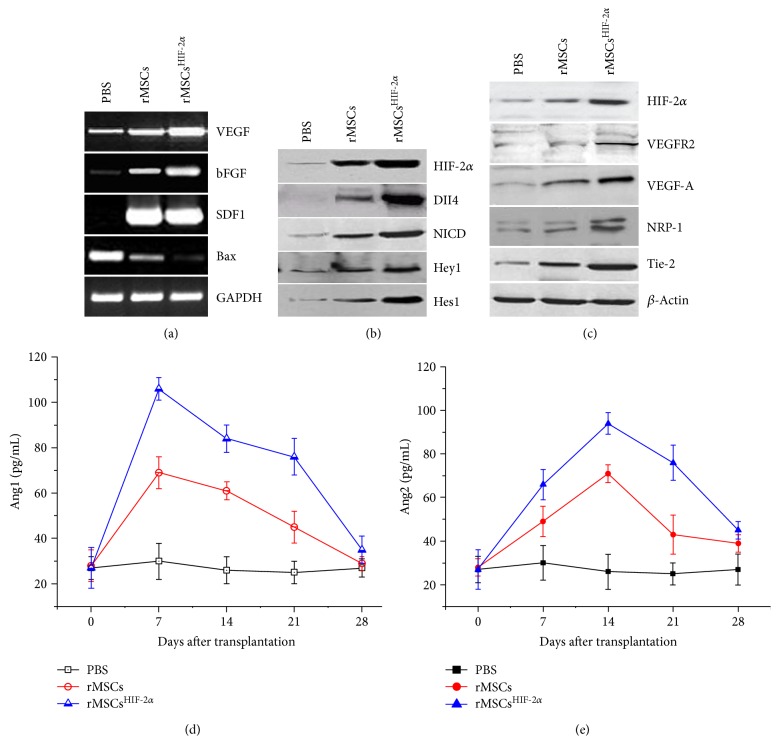
Cell transplantation increased the expression of growth factors and HIF-2*α* targeting molecules in the muscle tissue of the ischemic limb and cause dynamic change in serum angiopoietin. (a) Representative image of RT-PCR showing that the mRNA levels of VEGF, bFGF, and SDF1 increased and Bax mRNA level reduced 7 days after transplantation of rMSCs or rMSCs^HIF-2*α*^. (b and c) Representative images of Western blot showing that the expression of DII4, NICD, Hey1, Hes1, VEGFR2, VEGF-A, NRP-1, and Tie-2 increased 14 days after transplantation of rMSCs or rMSCs^HIF-2*α*^. (d) ELISA analysis of serum Ang1. (e) ELISA analysis of serum Ang2.

To investigate the molecular mechanism underlying the beneficial effect of rMSC^HIF-2*α*^ transplantation, we examined the expression of growth factors and HIF-2*α* targeting molecules in the muscle tissue of the ischemic limb and serum angiopoietin (Ang) levels. RT-PCR revealed that the mRNA levels of VEGF, bFGF, and SDF1 increased, while the Bax mRNA level reduced in both rMSC and rMSC^HIF-2*α*^ groups 7 days after the transplantation (Figure 4(a)). After 14 days of the transplantation, the protein levels of DII4, NICD, Hey1, Hes1, VEGFR2, VEGF-A, NRP-1, and Tie-2 increased substantially in the rMSC group and notably in the rMSC^HIF-2*α*^ group compared with the PBS group (Figures 4(b) and 4(c)). In addition, serum levels of Ang1 and Ang2 changed dynamically after the cell transplantation. Serum Ang1 and Ang2 levels peaked 7 and 14 days after the transplantation, respectively, and then reduced to the basal level gradually 28 days after the transplantation (Figures 4(d) and 4(e)). RMSC^HIF-2*α*^ transplantation increased the levels of all the tested molecules at a higher extent than rMSC transplantation (Figure 4). In rats with rMSC^HIF-2*α*^ transplantation, serum Ang1 (105 pg/mL) was markedly higher than Ang2 (65 pg/mL) 7 days after transplantation, and the peak serum Ang2 level (95 pg/mL) was slightly higher than Ang1 (82 pg/mL) on day 14 after transplantation. On day 28 after the transplantation, Ang1 and Ang2 were at similar levels in the 3 groups (Figures 4(d) and 4(e)).

**Figure 5:**
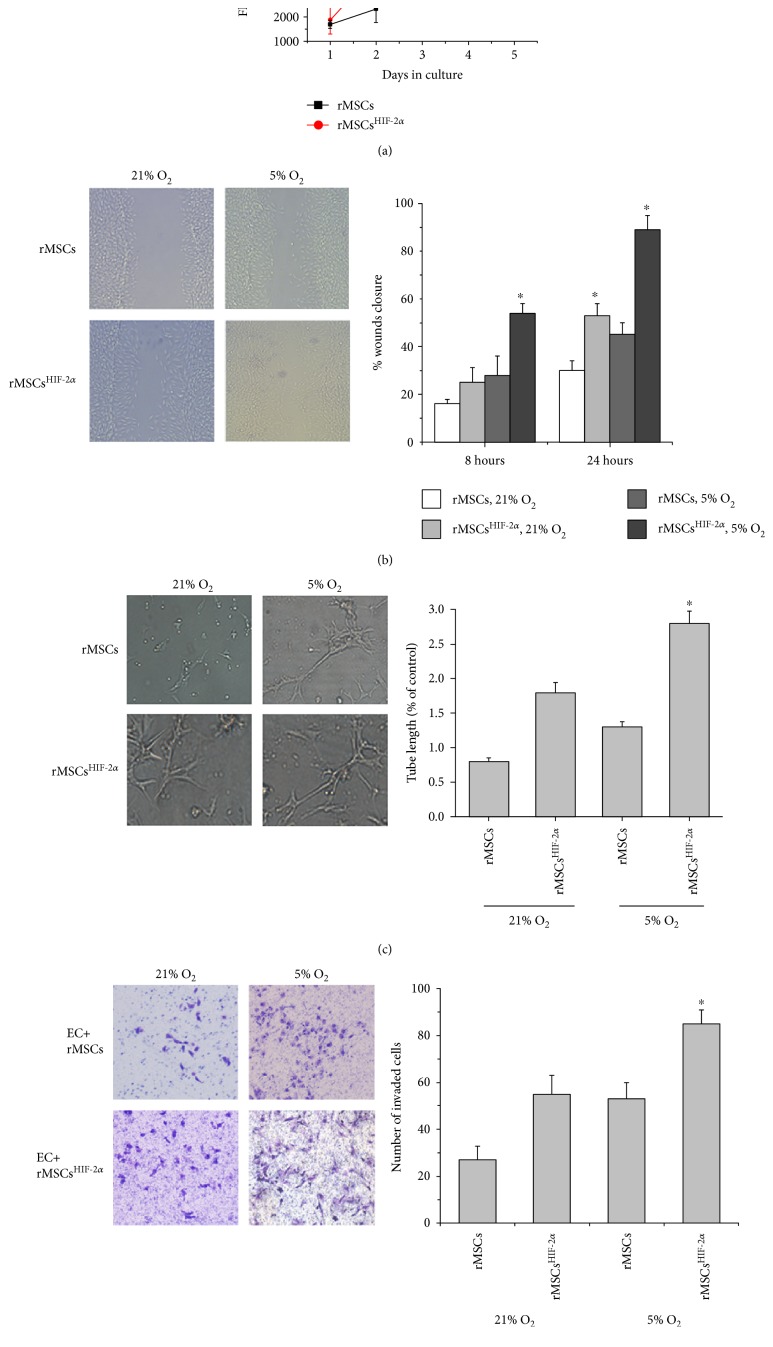
Effect of HIF-2*α* overexpression on rMSC behavior. (a) HIF-2*α* overexpression did not affect rMSC proliferation. (b) HIF-2*α* overexpression increased rMSC migration under hypoxia (5% O_2_) condition. The representative images of scratched cell layer were collected after 24 hours of incubation. ∗ indicates significant difference of the indicated group versus rMSCs group under the same condition, *P* < 0.05. (c) HIF-2*α* overexpression increased rMSC tube formation in matrigel under hypoxia (5% O_2_) condition. The representative images of tube formation in matrigel were collected after 24 hours of incubation. ∗ indicates significant difference of the indicated group versus rMSCs group under the same condition, *P* < 0.05. (d) Coculture with rMSCs^HIF-2*α*^ significantly increased the invasion of endothelial cells under hypoxia condition. The representative images of invaded endothelial cells were collected after 24 hours of incubation. ∗ indicates significant difference of the indicated group versus rMSCs group under the same condition, *P* < 0.05.

The effect of HIF-2*α* overexpression on rMSC behavior may also contribute to the rMSC^HIF-2*α*^-mediated attenuation of the ischemic damage. Overexpression of HIF-2*α* did not affect rMSC proliferation (Figure 5(a)), but increased rMSC migration significantly under both normoxia (21%) and hypoxia (5% O_2_) (*P* < 0.05, Figure 5(b)). The tube formation of rMSCs^HIF-2*α*^ in matrigel under hypoxia was increased significantly compared with rMSCs (*P* < 0.05, Figure 5(c)). In a transwell coculture system, rMSCs^HIF-2*α*^ induced the invasion of endothelial cells significantly under hypoxia (*P* < 0.05, Figure 5(d)).

## 4. Discussion

The current study demonstrated that transplantation of rMSCs^HIF-2*α*^ significantly improved blood perfusion and foot mobility in a rat hindlimb ischemia model. Similarly, Iwase and colleagues used a similar rat hindlimb ischemia model to show that transplantation of bone marrow rMSCs markedly improves blood perfusion and increases capillary density 21 days after transplantation [25]. In addition, transplantation of human bone marrow MSCs into the same type of rat ischemia model also improves blood perfusion and muscle performance 28 days after transplantation [26]. In the current study, we first compared the therapeutic potential of rMSCs^HIF-2*α*^ versus nongenetic modified rMSCs in a rat hindlimb ischemia model. We used allogenic MSCs and found no activation of immune system because of their lack or paucity of major histocompatibility complex class II and other immunoactive costimulatory molecules.

We found that the rMSC^HIF-2*α*^ transplantation significantly improved the blood perfusion in the ischemic limb compared to rMSC or PBS injection, and foot mobility of the ischemic limb was also significantly improved by rMSC^HIF-2*α*^ transplantation. RMSC^HIF-2*α*^ transplantation was also associated with increased host angiogenesis compared with rMSC transplantation. The number of vessel branches in the muscle tissue of the ischemic limb was significantly higher in the rMSCs^HIF-2*α*^ group than in the rMSCs group. These results indicate that genetic modification of rMSCs via overexpression of HIF-2*α* may improve short-term posttransplantation outcome in the rat hindlimb ischemia model.

The underlying mechanism associated with the superior posttransplantation outcome of rMSCs^HIF-2*α*^ can be mediated by both paracrine and autocrine pathways. We found that muscle tissues collected on posttransplantation day 7 or 14 contained higher levels of proangiogenic growth factors and cytokines in the rMSCs^HIF-2*α*^ group than in the rMSCs group, including VEGF and bFGF. Previous reports have shown that overexpression of HIF-1*α* in bone marrow MSCs significantly increases VEGF production [17]. HIF-1*α* has also been found to induce SDF1 in bone marrow-derived circulating progenitor cells and endothelial cells [27, 28]. Ang1 and Ang2 are vascular growth factors and play different roles in vascularization, thus promoting vessel maturation together. In the current study, serum Ang1 was markedly higher than Ang2 on day 7 after the transplantation; thus, the combined net effect of Ang1 and Ang2 was to promote angiogenesis at early posttransplantation time. Serum Ang1 level on posttransplantation day 7 was dramatically higher in the rMSCs^HIF-2*α*^ group (105 pg/mL) than in the rMSCs group (65 pg/mL). Therefore, rMSC^HIF-2*α*^ transplantation may induce substantially higher levels of proangiogenic growth factors and cytokines than rMSC transplantation at early stage after transplantation, which in turn could contribute to the significant improvement in blood perfusion and foot mobility at early stage after rMSC^HIF-2*α*^ transplant.

In contrast to the upregulation of proangiogenic factors, the level of the proapoptotic factor Bax was dramatically reduced in muscle tissue with rMSC^HIF-2*α*^ transplantation compared to rMSC transplantation. Thus, rMSCs^HIF-2*α*^ may be more resistant to apoptosis than rMSCs. The Notch signaling proteins DII4, NICD, Hey 1, and Hes1 were also markedly upregulated in muscle tissue with rMSC^HIF-2*α*^ transplantation compared with rMSC transplantation. Both HIF-1*α* and HIF-2*α* have been found to regulate Notch signaling in glioma stem cells [29]. In a mouse ischemia model, loss of DII4 damages arterial function and exacerbates ischemic damage, indicating that DII4 may play a key role in arterial functional recovery under ischemia [30]. Thus, in addition to induce proangiogenic factors, rMSC^HIF-2*α*^ transplantation may also enhance arterial function by upregulating Notch signaling and increase rMSCs^HIF-2*α*^ survival in the host by downregulating proapoptotic factors.

In the current study, results from the in vitro experiments also support that overexpression of HIF-2*α* could enhance posttransplantation outcome. HIF-2*α* overexpression did not affect rMSC proliferation under normoxic condition. However, under hypoxia, which resembles the in vivo condition in ischemic tissues, compared with rMSCs, rMSCs^HIF-2*α*^ were significantly more migratory, showed increased tube formation in matrigel, and significantly increased endothelial cell invasion.

## 5. Conclusions

Genetic modification of rMSCs via overexpression of HIF-2*α* improves posttransplantation outcomes in a rat hindlimb ischemia model. Transplantation of rMSCs^HIF-2*α*^ also induces neoangiogenesis at significantly higher extent than transplantation of the nongenetic modified rMSCs. The mechanisms underlying the beneficial effects appear to be associated with rMSCs^HIF-2*α*^-mediated upregulation of proangiogenic growth factors and cytokines.

## Supplementary Material

Supplementary Figure 1 Verification of the expression of HIF-2α in recombinant lentivirus. A. Image of agarose gel showing successful insertion of HIF-2α gene fragment in the vector. The insertion was confirmed by DNA sequencing of the recombinant vectors. The markers (2nd column) were 5 kb, 3 kb, 2 kb, 1.5 kb, 1 kb, 750 bp, 500 bp, 250 bp, 100 bp from top to bottom respectively. B. A representative image of Western blot showing that HIF-2α was expressed successfully. 293 cells transduced with recombinant lentivirus over-expressing HIF-2α or with empty lentivirus were harvest. Total proteins were extracted, separated on SDS-PAGE gel, and transferred to a PVDF membrane. The membrane was probed with anti-FLAG antibody. Lane 1 is the sample from 293 cells transduced with empty lentivirus and lance 3 is the sample from 293 cells transduced with the recombinant lentivirus. C. A representative fluorescence image showing 293 cells transduced with recombinant lentivirus. D. RT-PCR verification of HIF-2α expression in the 293 cells transduced with the recombinant lentivirus RT-PCT. The mRNA level of HIF-2α was dramatically increased in 293 cells transduced with the recombinant lentivirus compared to the controls.

## Abbreviations

Ang: Angiopoietin
Bax: Bcl-2-associated X protein
bFGF: Basic fibroblast growth factor
DII4: Delta-like ligand 4
HIF-1*α*: Hypoxia-inducible factors-1*α*
HIF-2*α*: Hypoxia-inducible factors-2*α*
LDPI: Laser Doppler perfusion image
MOI: Multiplicity of infection
NICD: Notch intracellular domain
NRP-1: Neuropilin 1
rMSCs: Rat bone marrow mesenchymal stem cells
rMSCs^HIF-2*α*^: Rat bone marrow MSCs overexpressing hypoxia-inducible factor 2*α*
SDF1: Stromal cell-derived factor 1
VEGF: Vascular endothelial growth factor
VEGFR2: Vascular endothelial growth factor receptor 2.
