# Supplementary figures and images for: Transplantation of Rat Mesenchymal Stem Cells Overexpressing Hypoxia-Inducible Factor 2α Improves Blood Perfusion and Arteriogenesis in a Rat Hindlimb Ischemia Model
Source: Stem Cells Int. 2017 Nov 7;2017:3794817. doi: 10.1155/2017/3794817 (PMC5697133; doi:10.1155/2017/3794817)

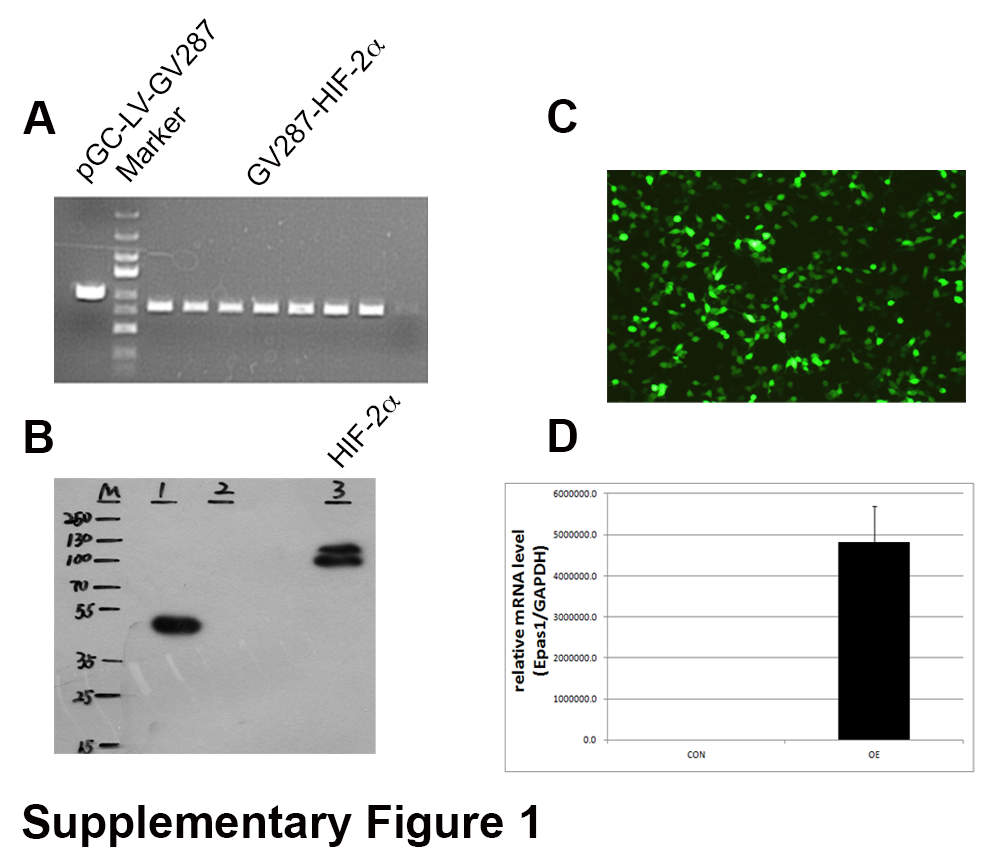

Supplement: Supplementary file 1 — Supplementary Figure 1 Verification of the expression of HIF-2α in recombinant lentivirus. A. Image of agarose gel showing successful insertion of HIF-2α gene fragment in the vector. The insertion was confirmed by DNA sequencing of the recombinant vectors. The markers (2nd column) were 5 kb, 3 kb, 2 kb, 1.5 kb, 1 kb, 750 bp, 500 bp, 250 bp, 100 bp from top to bottom respectively. B. A representative image of Western blot showing that HIF-2α was expressed successfully. 293 cells transduced with recombinant lentivirus over-expressing HIF-2α or with empty lentivirus were harvest. Total proteins were extracted, separated on SDS-PAGE gel, and transferred to a PVDF membrane. The membrane was probed with anti-FLAG antibody. Lane 1 is the sample from 293 cells transduced with empty lentivirus and lance 3 is the sample from 293 cells transduced with the recombinant lentivirus. C. A representative fluorescence image showing 293 cells transduced with recombinant lentivirus. D. RT-PCR verification of HIF-2α expression in the 293 cells transduced with the recombinant lentivirus RT-PCT. The mRNA level of HIF-2α was dramatically increased in 293 cells transduced with the recombinant lentivirus compared to the controls. [file 3794817.f1.tif]
